# Valve-Dependent Regional Heterogeneity of Wall Mechanics and Collagen Remodeling in Ascending Thoracic Aortic Aneurysms

**DOI:** 10.3390/ijms27062658

**Published:** 2026-03-14

**Authors:** Caroline Radner, Sandra Schmid, Moritz Sunderdiek, Yelyzaveta Sitnikova, Clara Hellmich, Linda Grefen, Maximilian Grab, Oliver Buchstab, Thomas Fabry, Nadja Sachs, Christian Hagl, Maximilian Pichlmaier, Sven Peterss, Joscha Buech

**Affiliations:** 1 Department of Cardiac Surgery, LMU University Hospital Munich, 81377 Munich, Germany; caroline.radner@med.uni-muenchen.de (C.R.); sandra.schmid22@web.de (S.S.); moritz.sunderdiek@med.uni-muenchen.de (M.S.); yelyzaveta.sitnikova@med.uni-muenchen.de (Y.S.); clara.hellmich@med.uni-muenchen.de (C.H.); linda.grefen@med.uni-muenchen.de (L.G.); maximilian.grab@med.uni-muenchen.de (M.G.); thomas.fabry@med.uni-muenchen.de (T.F.); christian.hagl@med.uni-muenchen.de (C.H.); maximilian.pichlmaier@med.uni-muenchen.de (M.P.); sven.peterss@med.uni-muenchen.de (S.P.); 2 University Aortic Centre Munich-LMU, LMU University Hospital Munich, 81377 Munich, Germany; 3 German Centre of Cardiovascular Research (DZHK), Partner Site Munich Heart Alliance, 80802 Munich, Germany; nadja.sachs@tum.de; 4 Institute of Pathology, Ludwig Maximilian University of Munich, 80802 Munich, Germany; oliver.buchstab@med.uni-muenchen.de; 5 Institute of Molecular Vascular Medicine, TUM Klinikum, Technical University Munich, 80802 Munich, Germany

**Keywords:** thoracic aortic aneurysm, bicuspid aortic valve, biomechanics, regional heterogeneity, wall shear stress

## Abstract

Ascending thoracic aortic aneurysm (ATAA) pathogenesis varies with aortic valve morphology, yet regional heterogeneity between inner curvature (IC) and outer curvature (OC) remains incompletely characterized. We hypothesized that regional differences between the outer and inner curvature of the ascending aorta are valve-morphology dependent and reflect distinct remodeling mechanisms in bicuspid versus tricuspid aortic valve-associated aortopathy. Ascending aortic tissue from 155 patients (69 tricuspid aortic valve [TAV], 68 bicuspid aortic valve [BAV], 18 non-aneurysmal heart transplantation [HTx] controls) underwent uniaxial tensile testing (*n* = 66), histological analysis, hydroxyproline assay, and reverse transcription quantitative PCR (RT-qPCR) for collagen (COL1A1, COL3A1, COL4A1, COL5A1, COL11A1) and elastin (ELN) genes. The OC was thinner than the IC in both TAV and BAV (*p* < 0.001), with no regional differences in HTx. TAV demonstrated increased OC stiffness (E-modulus 0.60 ± 0.31 vs. 0.43 ± 0.24 MPa, *p* = 0.004) with reduced failure strain (*p* = 0.013). BAV showed preserved stiffness but reduced OC extensibility (ε_max 56.5 ± 15.1% vs. 72.4 ± 21.7%, *p* < 0.001). BAV exhibited elevated OC collagen content (hydroxyproline OC/IC ratio 1.42, *p* = 0.048), whereas TAV showed reduced OC elastin area (*p* < 0.01). All collagen genes were upregulated at the OC in both TAV (all *p* < 0.001) and BAV (all *p* < 0.05), with COL11A1 showing the highest fold change (3.4-fold in TAV). ELN was reduced at the OC in TAV (*p* < 0.001) but unchanged in BAV. ATAAs exhibit distinct valve-dependent regional heterogeneities. The discordance between collagen gene expression and protein content suggests valve-specific differences in collagen regulation. These findings support distinct pathomechanisms and highlight the limitations of diameter-based risk stratification, motivating further investigation of regional wall assessment as a complement to current size criteria for surgical decision-making.

## 1. Introduction

Ascending thoracic aortic aneurysms (ATAAs) represent a heterogeneous group of pathologies with significant variability in pathogenesis, progression, and clinical outcomes [1]. Emerging evidence suggests that ATAA development is fundamentally influenced by underlying valve morphology and regional biomechanical factors within the ascending aorta [2].

Bicuspid aortic valve (BAV), the most common congenital cardiac anomaly affecting 1–2% of the population, is associated with substantially elevated risk of ascending aortic aneurysm formation [3]. BAV-associated aneurysms demonstrate distinct characteristics including earlier onset, more rapid progression, and frequently asymmetric dilatation patterns, suggesting a fundamentally different pathophysiological entity compared to tricuspid aortic valve (TAV)-associated aneurysms [4].

This heterogeneity extends to marked regional variations within the aortic wall [5]. The ascending aorta experiences complex hemodynamic forces that differ substantially between the inner (concave) and outer (convex) curvatures [6]. In BAV patients, eccentric flow patterns generate elevated wall shear stress particularly at the outer curvature, potentially creating distinct biomechanical microenvironments within the same aortic segment [7,8].

Previous studies have revealed differences in extracellular matrix composition between BAV and TAV aortas, including alterations in collagen/elastin ratios and collagen subtypes that regulate fibril diameter and tissue compliance [9,10]. However, a simultaneous, valve-stratified characterization of biomechanical, histological, and transcriptional OC/IC differences within a single well-characterized cohort have not been reported. The present study extends this literature by identifying a valve-specific discordance between collagen gene expression and protein content as a potential mechanistic differentiator between BAV- and TAV-associated aortopathy.

The present study aimed to characterize the biomechanical and molecular heterogeneity of ascending aortic aneurysms by comparing inner and outer curvature tissue properties in BAV and TAV patients using uniaxial tensile testing, quantitative gene expression analysis, and histological assessment to determine whether regional and valve-dependent differences correspond to features relevant for improved risk stratification and surgical decision-making. Current guidelines rely primarily on maximal aortic diameter to guide surgical timing; however, Kreibich et al. demonstrated that pre-dissection diameters were below 5.5 cm in 96% of TAV and below 5.0 cm in 76% of BAV patients who subsequently experienced type A aortic dissection, underscoring the clinical need for complementary wall-based risk markers beyond diameter [11].

## 2. Results

### 2.1. Patient Characteristics

Between 01/2023 and 12/2024, 155 patients were prospectively enrolled (TAV *n* = 69, BAV *n* = 68, HTx *n* = 18). Biomechanical analysis was performed in 66 patients with sufficient tissue for standardized specimen preparation (TAV *n* = 30, BAV *n* = 28, HTx *n* = 8). Paired OC and IC samples for histological analysis, collagen quantification by hydroxyproline assay, and gene expression analysis were available in all 155 patients. Baseline characteristics did not differ significantly between patients with and without biomechanical testing (*p* > 0.05, Supplementary Table S2). Mean age was 60 ± 11 years, 76.6% were male, and mean BMI was 26.9 ± 4.4 kg/m^2^. Cardiovascular risk factors included arterial hypertension (67.2%), hyperlipidemia (48.2%), diabetes mellitus (8.8%), and smoking (29.9%). Table 1 summarizes patient characteristics.

### 2.2. Biomechanical Properties

**Figure 1:**
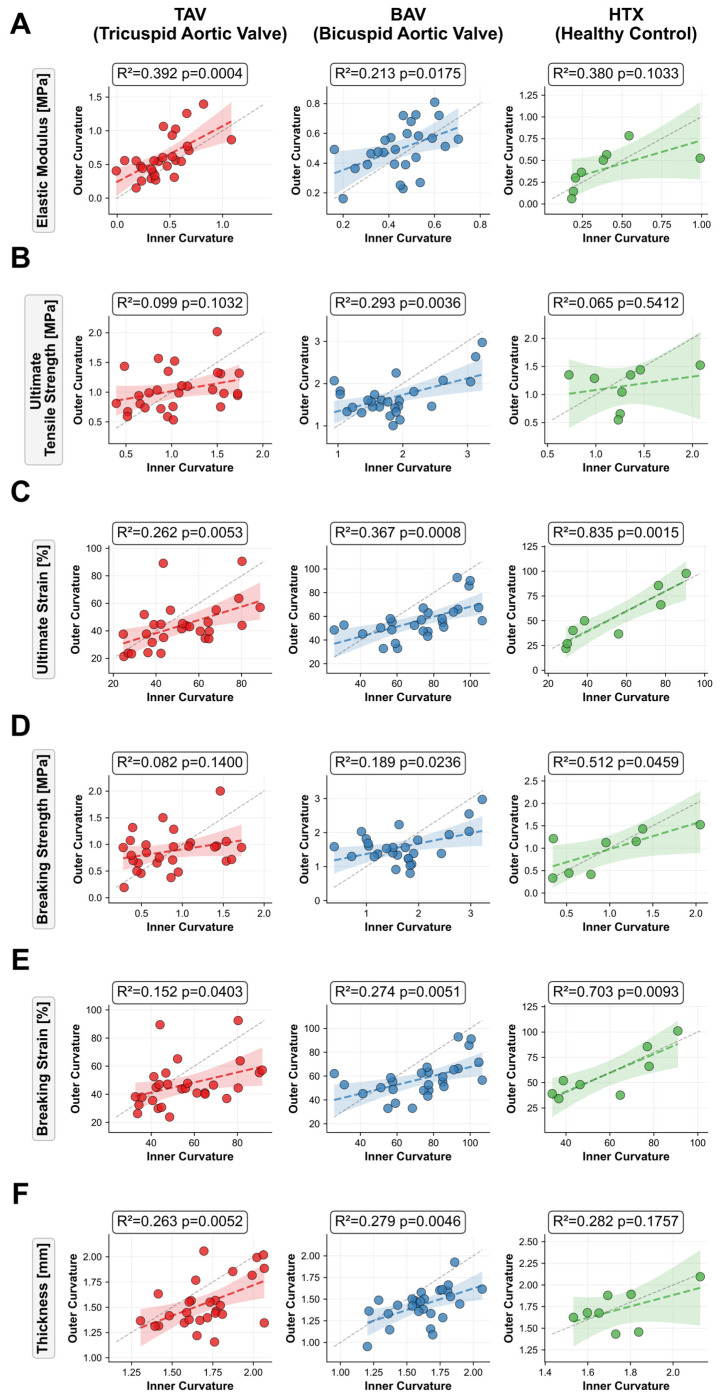
Regional correlation of biomechanical properties between outer and inner curvature. Scatter plots comparing paired measurements from outer curvature (*y*-axis) versus inner curvature (*x*-axis) for patients with tricuspid aortic valve (TAV, red), bicuspid aortic valve (BAV, blue), and non-aneurysmal controls from heart transplantation (HTx, green). Parameters shown: (**A**) elastic modulus, (**B**) ultimate tensile strength, (**C**) ultimate strain, (**D**) breaking strength, (**E**) breaking strain, and (**F**) wall thickness. Dashed line indicates line of identity. Linear regression lines with 95% confidence intervals are shown for each group. Coefficients of determination (R^2^) and *p*-values are provided per panel.

Uniaxial tensile testing revealed significant regional differences between OC and IC in aneurysmal tissue, while healthy controls showed homogeneous biomechanical properties (Table 2, Figure 1).

In TAV patients, the OC demonstrated significantly higher elastic modulus, lower ultimate strain, lower breaking strain, and reduced wall thickness compared to the IC (*p* < 0.05), whereas ultimate tensile strength and breaking strength did not differ between regions.

BAV patients exhibited more pronounced regional differences in extensibility, with the OC showing significantly lower ultimate strain, breaking strain, and thickness compared to the IC (all *p* < 0.001), while elastic modulus and strength parameters did not differ significantly.

HTx controls showed no significant differences between OC and IC for any biomechanical parameter. Across groups, OC samples from TAV aneurysms exhibited the highest elastic modulus and the lowest ultimate and breaking strain values. In contrast, OC samples from BAV aneurysms appeared to have higher ultimate and breaking strength compared with both TAV and HTx, while strain values at the OC were comparable between BAV and HTx. At the IC, ultimate and breaking strain values were higher in BAV than in TAV, and BAV also showed higher ultimate and breaking strength at the IC compared with both TAV and HTx. Elastic modulus at the IC was comparable across all three groups (Table 2).

OC and IC values within individual patients showed significant positive correlations across most parameters. In BAV, all parameters correlated significantly (R^2^ = 0.19–0.37, *p* < 0.05), in TAV correlations were significant for elastic modulus (R^2^ = 0.39, *p* < 0.001), strain parameters (R^2^ = 0.15–0.26, *p* < 0.05), and thickness (R^2^ = 0.26, *p* = 0.005), whereas HTx showed strong correlations for strain parameters (R^2^ = 0.70–0.84, *p* < 0.01) and a moderate correlation for breaking strength (R^2^ = 0.51, *p* = 0.046) (Figure 1).

### 2.3. Histology and Collagen Content

**Figure 2:**
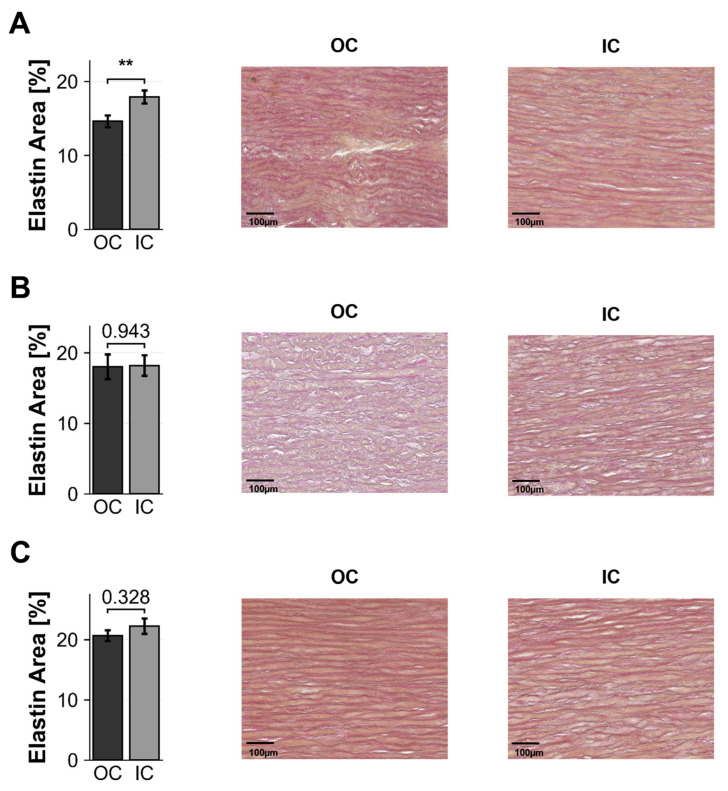
Regional elastin content. Quantification of elastin area (%) from Elastica van Gieson-stained sections comparing outer curvature (OC) versus inner curvature (IC) in (**A**) TAV, (**B**) BAV, and (**C**) HTx with representative histological images. Data presented as mean ± SD. Statistical comparison by paired *t*-test. ** *p* < 0.01. Scale bar = 100 µm.

Histological assessment revealed regional differences predominantly in BAV patients (Table 3, Figure 2). In BAV, the OC showed significantly higher cell density (574.1 ± 124.5 vs. 466.4 ± 126.7, *p* = 0.005) and lower media thickness (1.05 ± 0.18 vs. 1.17 ± 0.18 mm, *p* = 0.03) compared with the IC. Collagen content was higher at the OC (hydroxyproline OC/IC ratio 1.42 ± 0.21, *p* = 0.048), whereas elastin area did not differ between regions (18.0 ± 1.6% vs. 18.2 ± 1.5%, *p* = 0.943, Figure 2B). TAV patients showed a significantly lower elastin area at the OC compared with the IC (14.6 ± 0.8 vs. 17.9 ± 0.9%, *p* < 0.01, Figure 2A), but no significant regional differences in cell density, media thickness, or collagen content. HTx controls showed no significant regional differences for any histological parameter, but exhibited overall higher elastin area at both OC and IC (20.7 ± 0.9% and 22.2 ± 1.3%, respectively, Figure 2C) and lower collagen OC/IC ratios compared with aneurysmal aortas.

### 2.4. Gene Expression Analysis

**Figure 3:**
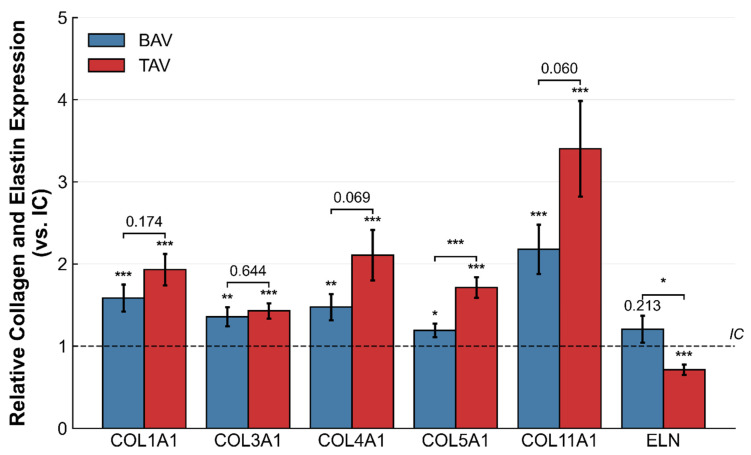
Regional gene expression of collagens and elastin. RT-qPCR analysis of COL1A1, COL3A1, COL4A1, COL5A1, COL11A1, and ELN expression at the outer curvature normalized to inner curvature (IC = 1, dashed line) in BAV (blue) and TAV (red). Asterisks above bars indicate significant difference from IC. Brackets indicate BAV vs. TAV comparison. No between-group difference reached significance after multivariable adjustment and FDR correction (Supplementary Table S3). Data presented as mean ± SEM. *p*-values were calculated using *t*-test. * *p* < 0.05, ** *p* < 0.01, *** *p* < 0.001.

RT-qPCR analysis revealed an upregulation of all studied collagen genes in the OC relative to the IC in TAV and BAV aneurysms (Figure 3). In TAV, all collagen genes (COL1A1, COL3A1, COL4A1, COL5A1, COL11A1) were significantly upregulated in the OC compared with the IC (*p* < 0.001), with COL11A1 showing the highest fold change (3.4-fold). Elastin (ELN) expression was significantly lower in the OC (*p* < 0.001). In BAV, COL1A1, COL3A1, COL4A1, COL5A1, and COL11A1 were similarly upregulated in the OC (*p* < 0.05), although fold changes were generally lower than in TAV. ELN expression did not differ significantly between regions (*p* = 0.213). In unadjusted analysis, regional differences (OC/IC ratio) were more pronounced in TAV than in BAV for COL5A1 (*p* < 0.001) and tended to be higher for COL11A1 (*p* = 0.060). ELN showed higher overall expression in BAV than in TAV (*p* < 0.05, unadjusted). After multivariable adjustment for age and aortic diameter, no between-group difference in OC/IC expression ratios reached statistical significance after Benjamini–Hochberg correction across all six genes (COL5A1: β = −0.404, 95% CI [−0.743, −0.065], *p*(adj) = 0.020, *p*(FDR) = 0.121; ELN: β = +0.304, 95% CI [−0.148, 0.756], *p*(adj) = 0.185). Full results are presented in Supplementary Table S3.

HTx controls showed no significant regional differences in collagen or elastin expression (Table 4).

## 3. Discussion

This study demonstrates significant regional variability in the biomechanical, histological, and molecular properties of ascending thoracic aortic aneurysms, with distinct patterns between BAV and TAV patients. Our finding that the inner curvature exhibits greater thickness, lower elastic modulus, and higher strain capacity compared to the outer curvature aligns with recent work by Salmasi et al., who reported that the outer aortic curve is stiffer and thinner than the inner curve in ATAAs [12]. In a related study, Chung et al. reported that the outer curvature exhibited greater energy loss during peel testing, although tangent modulus values showed a different regional pattern than observed in our uniaxial tensile data, likely reflecting differences in testing modality and the mechanical properties being assessed [13]. Importantly, the absence of regional differences in non-aneurysmal controls in our study suggests that this heterogeneity represents a pathological adaptation rather than normal anatomical variation. However, this interpretation should be treated with caution given the small HTx sample size (*n* = 8 for biomechanics) and the clinical heterogeneity of donor and recipient aortas, which may be subject to catecholamine-related or cardiomyopathy-associated remodeling that could obscure or mimic pathological regional patterns.

When comparing valve phenotypes, a distinct, previously described pattern emerged. The more pronounced regional heterogeneity in BAV compared to TAV is consistent with findings from Chung et al., who demonstrated that BAV aneurysms had higher delamination strength than TAV aneurysms, suggesting that BAV tissue may be biomechanically “healthier” and stronger despite dilatation and aneurysm formation [13]. They also found that BAV-associated aortopathy was characterized by higher delamination strength, while TAV aneurysms demonstrated greater energy loss during peel testing. Although delamination properties are not directly comparable to uniaxial tensile stiffness, these findings align with the concept that BAV tissue maintains greater structural integrity despite dilatation, which may explain why TAV patients in our cohort showed significant OC-IC differences in elastic modulus, while BAV did not.

The consistent intra-patient correlations between OC and IC values across all biomechanical parameters in BAV, albeit of modest effect size (R^2^ = 0.19–0.37), may suggest a more coordinated remodeling process, whereas the selective correlations in TAV could reflect more heterogeneous, regionally driven pathology.

Beyond these biomechanical differences, our molecular analysis revealed additional region-specific alterations that differed notably between valve phenotypes. In TAV, all collagen genes were markedly upregulated at the OC (all *p* < 0.001), yet hydroxyproline content did not differ between regions (OC/IC ratio 1.05, *p* = 0.639), indicating a clear discordance between transcriptional activity and net collagen protein accumulation. Several non-mutually exclusive mechanisms may account for this observation. First, the most pronounced transcriptional changes involved the regulatory collagens COL5A1 and COL11A1, which control fibril nucleation and diameter rather than bulk collagen mass [14]. A selective upregulation of these subtypes would alter collagen fibril architecture without substantially increasing total hydroxyproline content. Second, post-transcriptional mechanisms including translational regulation are known to uncouple mRNA and protein levels in aortic aneurysm tissue [15]. Third, increased collagen turnover with concurrent synthesis and MMP-mediated degradation, as demonstrated in abdominal aortic aneurysms, may contribute to elevated transcript levels without proportional protein accumulation in TAV tissue [16].

In contrast, BAV demonstrated a different pattern. Collagen gene upregulation at the OC was accompanied by a significant increase in hydroxyproline content (OC/IC ratio 1.42, *p* = 0.048), suggesting that the transcriptional response in BAV does translate into net collagen protein accumulation. This may reflect partial post-transcriptional attenuation of the collagen response. Alternatively, net hydroxyproline content may be primarily driven by the fibrillar collagens COL1A1 and COL3A1, which constitute the bulk of vascular collagen [17], whereas the regulatory collagens COL5A1 and COL11A1 contribute to qualitative changes in fibril architecture rather than total collagen mass [14]. It should be noted that the EvG-based collagen ratio in BAV (1.22, *p* = 0.136) did not reach significance, which may reflect the semi-quantitative nature of histomorphometric assessment compared to the biochemical specificity of the hydroxyproline assay.

These valve-specific patterns point to fundamentally different relationships between collagen gene expression and protein content. In TAV, the predominantly transcriptional response does not translate into net protein accumulation, possibly reflecting higher collagen turnover or degradation. In BAV, by contrast, elevated total collagen content at the OC in BAV indicates net collagen accumulation, although whether this reflects biomechanically effective fibrillar remodeling cannot be determined from hydroxyproline content alone, which provides no information on fibril diameter, cross-linking density, or spatial organization. This distinction may have functional implications. Net collagen accumulation in BAV could contribute to the preserved tensile strength observed biomechanically. Conversely, the failure to accumulate collagen protein in TAV despite elevated transcription may explain OC stiffening without proportional strengthening. However, the relationship between total collagen content and fibrillar architecture remains unresolved with the present data, and future studies incorporating collagen subtype-specific protein quantification and assessment of fibril ultrastructure will be needed to further delineate these valve-specific remodeling pathways [18].

While previous studies have examined biomechanical regional variation and layer-specific ECM composition, the circumferential distribution of collagen and elastin gene expression between outer and inner curvature has not been systematically characterized [12,19,20]. The pronounced upregulation of these regulatory collagens at the outer curvature suggests region-specific transcriptional remodeling that may precede or accompany the observed biomechanical differences. The finding that TAV showed more pronounced collagen upregulation than BAV in unadjusted analyses is consistent with distinct pathophysiological mechanisms. However, after adjustment for age and aortic diameter, these between-group differences did not reach statistical significance, and this observation should therefore be considered exploratory.

These molecular and microstructural changes, however, should be interpreted in the context of the local hemodynamic environment. The inner and outer curvature are exposed to distinct flow patterns, particularly in BAV, where eccentric systolic jets preferentially impinge on the outer curvature and generate regionally elevated wall shear stress [21,22]. This sustained hemodynamic loading may drive the collagen-rich stiffening observed at the OC, whereas the IC appears to undergo compensatory thickening with preserved distensibility. Salmasi et al. reported that the outer aortic curve is thinner and stiffer than the inner curve and may be more prone to dissection propagation, while the inner curve is relatively protected, which aligns with our finding that OC tissue shows higher stiffness but comparable strength, a combination that may predispose to structural failure modes [12].

Considered collectively, these biomechanical, molecular and hemodynamic findings point towards potential implications for further clinical evaluation. Our data support the notion that aortic diameter alone inadequately captures dissection risk [23]. The regional heterogeneity observed in aneurysmal but not healthy tissue suggests that monitoring regional wall properties could improve risk stratification. Kreibich et al. reported that predissection ascending aortic diameters were below 5.5 cm in 96% of TAV patients and below 5.0 cm in 76% of BAV patients who later experienced aortic dissection, highlighting the limitations of current size-based criteria [11]. These results suggest that a more regionally resolved assessment of wall integrity may help refine surgical timing especially in patients with borderline aortic dimensions.

### Limitations

Several limitations should be acknowledged. The crosshead velocity of 10 mm/min is at the upper end of the quasi-static rates commonly used in the literature (2–10 mm/min) which may affect absolute values through viscoelastic effects. However, relative comparisons between regions are likely to remain valid. Specimens were tested at room temperature without continuous hydration, which may affect absolute mechanical values due to tissue dehydration. Since all specimens were processed and tested under identical ambient conditions, internal comparisons remain unaffected. Testing was performed in circumferential orientation only as tissue dimensions did not permit standardized longitudinal specimen preparation. Given the known anisotropy of aortic tissue, longitudinal testing may reveal additional regional differences not captured here. Nevertheless, circumferential properties are of particular interest because they provide the primary resistance to wall tension in the ascending aorta under physiological pressures. The HTx control group was small (*n* = 8) due to the inherent scarcity of donor tissue, thereby limiting statistical power for this comparison. The HTx group served as a non-aneurysmal reference rather than a physiological baseline, as donor and recipient aortas may be affected by cardiomyopathy-associated aortopathy, catecholamine exposure, or other subclinical pathology. The absence of clinical data for this group precludes assessment of cardiovascular risk factors. Subgroup analyses by valve pathology (stenosis vs. regurgitation) and BAV fusion type (R-L, R-N, L-N) were not performed due to insufficient sample sizes, particularly for biomechanical testing, although these factors influence hemodynamic profiles and may differentially affect regional wall properties. TAV patients had significantly larger aortic diameters than BAV patients (54.8 vs. 50.9 mm, *p* < 0.001) and were significantly older (64 ± 10 vs. 56 ± 11 years, *p* < 0.001), consistent with established earlier surgical thresholds for BAV, but age- and diameter-related confounding of between-group comparisons cannot be excluded. Medication data, including statins and antihypertensives that may affect ECM metabolism, were not systematically collected. Finally, this single-center study may not fully represent the heterogeneity of ATAA populations, underscoring the need for multicenter validation in broader cohorts. Despite these limitations, the consistency of regional patterns across patient groups strengthens the internal validity of our findings. Direct protein-level quantification of individual fibrillar collagen subtypes (COL5A1, COL11A1) from tissue homogenates is technically challenging due to their high insolubility and extensive crosslinking, which limits efficient extraction for Western blot or ELISA-based approaches; total collagen content was therefore assessed via the hydroxyproline assay. Future studies should address protein-level quantification of individual collagen subtypes to complement the mRNA and total hydroxyproline data presented here. Additionally, profiling of MMPs and matricellular proteins such as thrombospondin-1 and osteopontin would provide important mechanistic context regarding collagen turnover and remodeling regulation. Immune cell characterization, including macrophage and T-cell infiltration, in OC versus IC tissue represents a further avenue that was beyond the scope of the present investigation but may contribute to understanding the inflammatory drivers of regional wall remodeling.

## 4. Materials and Methods

### 4.1. Patient Population and Clinical Data

Patients (≥18 years) who underwent elective aortic surgery between January 2023 and December 2024 were prospectively enrolled after obtaining written informed consent. All identifiable patient data were pseudonymized prior to analysis and stored in a password-protected institutional database accessible exclusively to study personnel. Written informed consent was obtained from all participants prior to tissue collection. HTx control tissue was collected anonymously under institutional approval. No individual patient data were available for this group. Patients were stratified into two subgroups based on aortic valve morphology, either TAV or BAV. Non-aneurysmal aortic tissue obtained from heart transplant donors and recipients (HTx) served as controls. Clinical data, including age, sex, body mass index (BMI), and cardiovascular risk factors (arterial hypertension, hyperlipidemia, diabetes mellitus, smoking status) were obtained from electronic medical records. Maximal aortic diameter was determined from the most recent preoperative contrast-enhanced computed tomography angiography (CTA) using centerline reconstruction (Visage Client 7.1, Visage Imaging GmbH, Berlin, Germany). The study was approved by the Ethics Committee of Ludwig-Maximilians-Universität München (Protocol No.22-0384) and conducted in accordance with the Declaration of Helsinki.

### 4.2. Sample Collection and Processing

During surgery, a complete aortic ring was excised 3–5 cm above the sinotubular junction from the mid-section of the ascending aorta. Anatomical orientation was marked using titanium clips. Specimens were immediately transferred into Dulbecco’s Modified Eagle Medium (DMEM, Thermo Fisher Scientific, Waltham, MA, USA) at 4 °C and processed within six hours.

**Figure 4:**
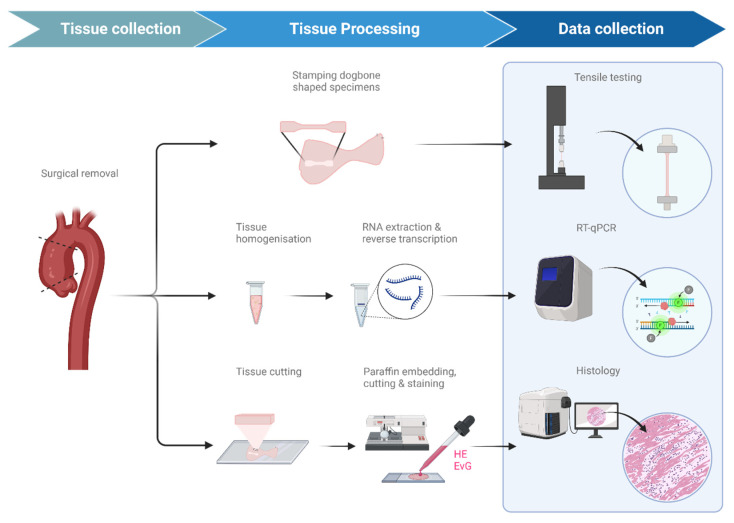
Experimental workflow. Outer and inner curvature tissue from ascending aortic surgical specimens were sampled and divided for three analyses. Dogbone specimens underwent uniaxial tensile testing, homogenized tissue was processed for RT-qPCR of collagen and elastin genes, and paraffin-embedded sections were stained with HE and Elastica van Gieson for histological evaluation.

Aortic tissue was systematically processed for three analytical modalities (Figure 4):

1. For biomechanical analysis, dogbone-shaped specimens (gauge length: 12 mm, gauge width: 2 mm; DIN 53504-S3) were stamped from both the outer curvature (OC) and the inner curvature (IC) in circumferential orientation and subjected to uniaxial tensile testing.
2. For molecular analysis, tissue samples from each curvature were snap-frozen in RNA preservation solution (RNAlater™, Thermo Fisher Scientific, Waltham, MA, USA) and stored at −80 °C until RNA extraction or collagen quantification.
3. For histological analysis, circumferentially oriented tissue sections from both curvatures were formalin-fixed and paraffin-embedded for subsequent staining.

### 4.3. Uniaxial Tensile Testing

Uniaxial tensile testing was performed using a zwickiLine universal testing machine (ZwickRoell GmbH, Ulm, Germany) with at least two replicates per curvature. Testing was performed at room temperature under ambient conditions. Specimens were kept moist with physiological saline prior to mounting. Adventitial tissue was carefully removed by sharp dissection prior to specimen preparation. Specimen thickness was measured at three locations using a digital thickness gauge (Mitutoyo Corporation, Kawasaki, Japan) and averaged. Specimens were mounted between mechanical screw-action grips and pre-loaded to 0.1 N to establish a uniform baseline. Tensile testing was conducted at a constant crosshead velocity of 10 mm/min until specimen failure.

Force and displacement data were continuously recorded and converted to engineering stress (σ = F/A_0_) and strain (ε = ΔL/L_0_) based on initial specimen dimensions. From the resulting stress–strain curves, the following parameters were derived and compared between OC and IC specimens including elastic modulus (Et), ultimate tensile strength (σM), strain at maximum stress (εM), breaking strength (σB), and breaking strain (εB).

### 4.4. Gene Expression Analysis (RT-qPCR)

For gene expression analysis, frozen tissue samples were thawed on ice and adventitial tissue was carefully removed by sharp dissection. Total RNA was extracted from OC and IC using the miRNeasy Mini Kit (Qiagen, Hilden, Germany) according to the manufacturer’s protocol. RNA concentration and purity were assessed using a NanoDrop spectrophotometer (Thermo Fisher Scientific), and samples with 260/280 ratios between 1.8–2.1 were used for downstream analysis. Complementary DNA (cDNA) was synthesized using the High-Capacity RNA-to-cDNA Kit (Applied Biosystems, Waltham, MA, USA). RT-qPCR was performed using TaqMan Fast Advanced Master Mix (Applied Biosystems) with pre-validated gene-specific TaqMan primers (Supplementary Table S1). RPLP0 served as the reference gene. Relative gene expression was calculated using the 2^−ΔΔCt^ method [24].

### 4.5. Collagen Content (Hydroxyproline Assay)

Hydroxyproline content was quantified in tissue samples from OC and IC using a colorimetric assay kit (Sigma-Aldrich, St. Louis, MO, USA) according to the manufacturer’s protocol. Absorbance was measured at 560 nm and concentrations were normalized to tissue wet weight.

### 4.6. Histological Analysis

Tissue samples from the OC and the IC were formalin-fixed and paraffin-embedded. Sections (4 µm) were stained with hematoxylin–eosin (HE) for cell nuclei quantification and Elastica van Gieson (EvG) for elastin and collagen content. Digital images were acquired using a slide scanner (Aperio GT 450 DX, Leica Biosystems, Nussloch, Germany) at 40× magnification and analyzed using QuPath software (Version 0.6.0) [25]. For each section, four randomly selected regions of interest (2000 × 2000 pixels) were evaluated. Media thickness was measured at four locations per section. Elastin and collagen content were quantified using threshold-based segmentation with a manually trained pixel classifier. Cell nuclei density was expressed as nuclei per ROI. All histological analyses were performed in duplicate by two independent, experienced observers, both blinded to sample identity and clinical information. For each parameter, values from both observers were averaged for statistical analysis.

### 4.7. Statistical Analysis

Statistical analysis was performed using Python (version 3.11) with SciPy, NumPy, and Pandas libraries. Continuous variables are expressed as mean ± standard deviation (SD) and categorical variables as *n* (%). Gene expression data are presented as mean ± standard error of the mean (SEM). When multiple specimens per location were obtained from a single patient, values were averaged. Normality was assessed using Shapiro–Wilk tests. Paired comparisons between OC and IC were performed using paired *t*-tests. Since OC and IC specimens are obtained from the same aortic ring of the same patient, aortic diameter does not differ between paired specimens by design and is therefore not expected to confound within-patient regional comparisons. Comparisons between independent groups (BAV vs. TAV vs. HTx) were analyzed using unpaired *t*-tests or Mann–Whitney U tests, as appropriate. Correlations between OC and IC biomechanical parameters were assessed using simple linear regression and reported as coefficients of determination (R^2^). The associated *p*-values reflect the significance of the regression slope (equivalent to testing whether the Pearson correlation coefficient differs from zero). Categorical variables were compared using Chi-square test. To assess confounding by age and aortic diameter on between-group (BAV vs. TAV) differences in OC/IC expression ratios, multivariable linear regression was performed for each of the six RT-qPCR outcomes, with valve morphology (BAV = 1, TAV = 0), age, and maximal aortic diameter as independent variables. Full results are provided in Supplementary Table S3. *p*-values were adjusted for multiple comparisons using the Benjamini–Hochberg method. *p*-values < 0.05 were considered statistically significant.

## 5. Conclusions

This study demonstrates that ascending thoracic aortic aneurysms exhibit significant regional heterogeneity between the outer and inner curvature, which is absent in non-aneurysmal aortic tissue. The inner curvature is characterized by greater thickness, higher distensibility, and lower stiffness compared with the outer curvature, with BAV patients showing more pronounced and consistent regional differences than TAV patients. Regional differences in collagen and elastin gene expression, particularly the upregulation of regulatory collagens COL5A1 and COL11A1 at the outer curvature, suggest active transcriptional remodeling that translates into net collagen protein accumulation in BAV but not in TAV, pointing to valve-specific differences in post-transcriptional collagen regulation. However, between-group differences in OC/IC expression ratios did not reach statistical significance after multivariable adjustment and FDR correction, and these observations should therefore be considered exploratory pending independent validation in cohorts with matched baseline characteristics. These findings support the presence of distinct pathophysiological mechanisms in BAV versus TAV aortopathy. Together, these structural, mechanical, and molecular patterns indicate that ATAA behavior varies regionally rather than uniformly and highlight the limitations of diameter-based risk stratification. Therefore, these findings encourage further investigation of regional biomechanical assessment as a complement to diameter-based risk stratification for acute aortic events.

## Figures and Tables

**Table 1:** Patient characteristics. Data are presented as mean ± SD or *n* (%). *p*-values were calculated using unpaired *t*-tests for continuous variables and Chi-square tests for categorical variables. Bold values indicate statistical significance.

Variable	TAV	BAV	*p*-Value
Age (years)	64 ± 10	56 ± 11	**<0.001**
Sex (male), *n* (%)	53 (76.8%)	52 (76.5%)	1.0
BMI (kg/m^2^)	27.1 ± 4.9	26.7 ± 3.8	0.538
Aortic diameter (mm)	54.8 ± 7.7	50.9 ± 5.1	**<0.001**
Hypertension, *n* (%)	49 (71.0%)	43 (63.2%)	0.632
Hyperlipidemia, *n* (%)	34 (49.3%)	32 (47.1%)	0.864
Diabetes mellitus, *n* (%)	9 (13.0%)	3 (4.4%)	0.131
Smoking, *n* (%)	26 (37.7%)	14 (20.6%)	0.062

**Table 2:** Biomechanical properties comparing outer and inner curvature. Elastic modulus, ultimate tensile strength (σ_max), ultimate strain (ε_max), breaking strength (σ_break), breaking strain (ε_break), and wall thickness at OC versus IC in TAV, BAV, and HTx. Data presented as mean ± SD. *p*-values were calculated using paired *t*-test. Bold values indicate statistical significance.

Group	Parameter	OC (Mean ± SD)	IC (Mean ± SD)	*p*-Value
TAV	E-Modulus (MPa)	0.60 ± 0.31	0.43 ± 0.24	**0.004**
	σ_max (MPa)	1.03 ± 0.35	1.07 ± 0.42	0.662
	ε_max (%)	43.57 ± 17.30	51.17 ± 18.39	**0.013**
	σ_break (MPa)	0.87 ± 0.37	0.86 ± 0.46	0.972
	ε_break (%)	46.76 ± 16.44	55.56 ± 17.90	**0.020**
	Thickness (mm)	1.54 ± 0.25	1.71 ± 0.21	**<0.001**
BAV	E-Modulus (MPa)	0.50 ± 0.16	0.45 ± 0.13	0.143
	σ_max (MPa)	1.67 ± 0.44	1.84 ± 0.62	0.065
	ε_max (%)	56.49 ± 15.07	72.40 ± 21.66	**<0.001**
	σ_break (MPa)	1.58 ± 0.49	1.69 ± 0.69	0.361
	ε_break (%)	57.68 ± 15.29	73.39 ± 21.50	**<0.001**
	Thickness (mm)	1.43 ± 0.20	1.62 ± 0.21	**<0.001**
HTx	E-Modulus (MPa)	0.41 ± 0.24	0.39 ± 0.27	0.641
	σ_max (MPa)	1.15 ± 0.37	1.30 ± 0.39	0.383
	ε_max (%)	53.16 ± 27.57	53.79 ± 24.79	0.945
	σ_break (MPa)	0.95 ± 0.48	0.96 ± 0.59	0.742
	ε_break (%)	58.00 ± 24.38	58.26 ± 22.13	0.844
	Thickness (mm)	1.72 ± 0.23	1.75 ± 0.18	0.945

**Table 3:** Histological and biochemical comparison between outer and inner curvature. Cell count, media thickness, and collagen content (EvG staining and hydroxyproline assay) comparing OC versus IC in TAV, BAV, and HTx. Collagen ratios expressed as OC/IC. Data presented as mean ± SD. *p*-values were calculated using paired *t*-test. Collagen ratios were not formally compared between valve groups. Between-group observations are reported descriptively. Bold values indicate statistical significance.

Variable	TAV (Mean ± SD)	BAV (Mean ± SD)	HTx (Mean ± SD)
Cells, *n*	OC 585.7 ± 181.4	OC 574.1 ± 124.5	OC 527.0 ± 178.6
	IC 553.1 ± 221.1	IC 466.4 ± 126.7	IC 553.4 ± 77.1
	*p* = 0.451	***p*** **= 0.005**	*p* = 0.714
Media Thickness (mm)	OC 1.22 ± 0.16	OC 1.05 ± 0.18	OC 1.35 ± 0.14
	IC 1.30 ± 0.19	IC 1.17 ± 0.18	IC 1.39 ± 0.22
	*p* = 0.075	***p*** **= 0.03**	*p* = 0.639
Collagen Ratio (EvG, OC/IC)	1.01 ± 0.09	1.22 ± 0.14	1.07 ± 0.09
	*p* = 0.909	*p* = 0.136	*p* = 0.363
Collagen Ratio (Hydroxyproline OC/IC)	1.05 ± 0.12	1.42 ± 0.21	0.88 ± 0.27
	*p* = 0.639	***p*** **= 0.048**	*p* = 0.729

**Table 4:** Regional collagen and elastin gene expression in non-aneurysmal controls (OC/IC ratio). RT-qPCR analysis of collagen (COL1A1, COL3A1, COL4A1, COL5A1, COL11A1) and elastin (ELN) expression at OC relative to IC in HTx. Data presented as mean ± SEM. FC = fold change. *p*-values were calculated using *t*-test.

Gene	FC (Mean ± SEM)	*p*-Value
COL1A1	1.08 ± 0.28	0.252
COL3A1	1.01 ± 0.20	0.938
COL4A1	1.21 ± 0.34	0.109
COL5A1	1.11 ± 0.31	0.376
COL11A1	0.85 ± 0.54	0.305
ELN	1.19 ± 0.52	0.303

## Data Availability

The data presented in this study are available on reasonable request from the corresponding author. The data are not publicly available due to patient privacy and ethical restrictions.

## Supplementary Materials

The following supporting information can be downloaded at: https://www.mdpi.com/article/10.3390/ijms27062658/s1.

## Abbreviations

The following abbreviations are used in this manuscript:

ATAA	Ascending thoracic aortic aneurysm
BAV	Bicuspid aortic valve
TAV	Tricuspid aortic valve
HTx	Heart transplantation
OC	Outer curvature
IC	Inner curvature
ECM	Extracellular matrix
COL1A1	Collagen type I alpha 1
COL3A1	Collagen type III alpha 1
COL4A1	Collagen type IV alpha 1
COL5A1	Collagen type V alpha 1
COL11A1	Collagen type XI alpha 1
ELN	Elastin
RT-qPCR	Reverse transcription quantitative polymerase chain reaction
cDNA	Complementary DNA
RPLP0	Ribosomal protein lateral stalk subunit P0
EvG	Elastica van Gieson
HE	Hematoxylin–eosin
CTA	Computed tomography angiography
DMEM	Dulbecco’s Modified Eagle Medium
BMI	Body mass index
SD	Standard deviation
SEM	Standard error of the mean
MMP	Matrix metalloproteinase
WSS	Wall shear stress
