# Supplementary material for: Valve-Dependent Regional Heterogeneity of Wall Mechanics and Collagen Remodeling in Ascending Thoracic Aortic Aneurysms
Source: Int J Mol Sci. 2026 Mar 14;27(6):2658. doi: 10.3390/ijms27062658 (PMC13026728; doi:10.3390/ijms27062658)
Supplement: Supplementary file 1 [file ijms-27-02658-s001.zip › ijms-4184017-supplementary.pdf]

International Journal of *Molecular Sciences*

MDPI

Article

Valve-Dependent Regional Heterogeneity of Wall Mechanics and Collagen Remodeling in Ascending Thoracic Aortic Aneurysms

Caroline Radner [1,2,3,#] Sandra Schmid [1,2,#], Moritz Sunderdiek [1,2,3], Yelyzaveta Sitnikova [1,2,3], Clara Hellmich [1,2], Linda Grefen [1,2,3], Maximilian Grab [1,2,3], Oliver Buchstab [4], Thomas Fabry [1,2], Nadja Sachs [3,5], Christian Hagl [1,2,3], Maximilian Pichlmaier [1,2], Sven Peterss [1,2] and Joscha Buech [1,2,3]

[1] Department of Cardiac Surgery, LMU University Hospital Munich, Munich, Germany
[2] University Aortic Centre MunichLMU, LMU University Hospital Munich, Munich, Germany
[3] German Centre of Cardiovascular Research (DZHK), Partner Site Munich Heart Alliance, Munich, Germany
[4] Institute of Pathology, Ludwig Maximilian University of Munich, Munich, Germany
[5] Institute of Molecular Vascular Medicine, TUM Klinikum, Technical University Munich, Munich, Germany
[#] Authors contributed equally

Supplementary Table S1: TaqMan™ Gene Expression probes used for RT-qPCR.

Gene	Assay ID
COL1A1	Hs01651334_m1
COL3A1	Hs00943809_m1
COL4A1	Hs00266237_m1
COL5A1	Hs00609133_m1
COL11A1	Hs01097664_m1
ELN	Hs00355783_m1

Supplementary Table S2: Baseline characteristics of patients with and without biomechanical testing.

Variable	TAV			BAV		
	mech	non-mech	p-value	mech	non-mech	p-value
Age (years)	66±7	62±12	0.133	57±8	55±12	0.524
Sex (male), n (%)	21 (70%)	32 (82.1%)	0.374	20 (71.4%)	32 (80%)	0.596
BMI (kg/m²)	27.5±4.6	26.9±5.3	0.649	26.8±4	26.6±3.8	0.817
Aortic diameter (mm)	54.8±6.2	54.7±9	0.946	50.3±4.5	51.2±5.5	0.499
Hypertension, n (%)	24 (80%)	26 (64.1%)	0.124	16 (57.1%)	27 (67.5%)	0.538
Hyperlipidemia, n (%)	12 (40%)	22 (56.4%)	0.327	14 (50%)	18 (45%)	0.873
Diabetes mellitus, n (%)	1 (3.3%)	8 (20.5%)	0.091	1 (3.6%)	2 (5%)	1.0
Smoking, n (%)	8 (26.7%)	18 (46.2%)	0.192	7 (25%)	8 (20%)	0.848

Mech = patients with biomechanical testing; no-mech = patients without biomechanical testing. Data

Gene	β (BAV vs. TAV)	95% CI	p(multivariable)	p(multivariable, FDR)	p(unadjusted)
COL1A1	−0.040	[−0.661, 0.582]	0.900	0.900	0.174
COL3A1	−0.150	[−0.604, 0.303]	0.510	0.765	0.644
COL4A1	−0.384	[−1.115, 0.347]	0.298	0.596	0.069
COL5A1	−0.404	[−0.743, −0.065]	0.020	0.121	0.001
COL11A1	−0.244	[−1.464, 0.975]	0.692	0.830	0.060
ELN	+0.304	[−0.148, 0.756]	0.185	0.554	0.011

are mean ± SD or n (%). P-values were calculated using t-test.

Supplementary Table 3. Multivariable linear regression of OC/IC expression ratios for valve morphology (BAV vs. TAV), adjusting for age and maximal aortic diameter.

Multivariable linear regression was performed for all six RT-qPCR outcomes in 137 aneurysmal patients with complete data (TAV n=69, BAV n=68). The OC/IC expression ratio was modeled as the dependent variable with valve morphology (BAV=1, TAV=0), age, and maximal aortic diameter as independent variables. β coefficients reflect the difference in OC/IC ratio for BAV relative to TAV after adjustment. p(multivariable, FDR): Benjamini-Hochberg correction applied to multivariable p-values. A negative β indicates a lower OC/IC ratio in BAV compared to TAV.
